# Chicago Academy of Medical Sciences

**Published:** 1860-04

**Authors:** Walter Hay

**Affiliations:** Sec’y.


### CHICAGO ACADEMY OF MEDICAL SCIENCES.

(Reported for "Medical Examiner," by WALTER HAY, M. D., Sec'y.

This Academy assembled in their new rooms, No. Washington Street, and being called to order by the President, the proceedings of the last meeting were read, accepted, and ordered to be recorded. The Secretery read a report from the Council Committee appointed to make purchases of furniture and fittings for the new rooms, submitting therewith vouchers for moneys expended, which was also accepted and ordered on file.

Dr. Hamill read a paper upon Sulphate of Quinine and some of its effects.

Dr. Ingalls thought the remedy was usually given in too large doses, thereby proving a local irritant, and developing any latent typhoid tendency.

Dr. Hay agreed with the gentleman in recommending the use of small doses of quinine frequently repeated, in intermittents; did not consider it a local irritant.

Dr. Rauch did not believe quinine to be a local irritant, but thought that it must necessarily be absorbed in order to produce its physiological effects.

Dr. O. Smith thought that the remedy in question must act by means of absorption upon the nervous system as a stimulant contracting the arteries, and increasing the fulness and hardness of the pulse.

Dr. Holmes asked for information regarding the efficacy of small doses, and the actual amount necessary to be administered.

Dr. Wickersham thought 14 to 16 grains the smallest quantity necessary to be introduced into the system in order to overcome an attack of intermittent.

Dr. McAllister related the treatment of two cases, illustrating the efficacy of small doses of quinine preceded by a mercurial cathartic, after large doses had proved inefficient.

Dr. Byford thought that in Dr. McAllister's cases above mentioned, much allowance was to be made for the influence of change of climate and season, and related a summary of the changes which had taken place in the mode of using quinine, during a period of twenty years within his experience; he considered the remedy to possess, in addition to its other qualities, diaphoretic properties, and favored its administration in large doses of three to five grains. He considered that the absence of uric acid in the urine to be attributable rather to the diminution in the waste of tissue than to defective elimination. He believed that quinine was an irritant relatively, frequently as much so in small as in large doses, and that its effects were generally too evanescent to prove injurious. He had seen no harm, nor any good result from its use in the treatment of typhoid fever, and believed, that in order to the production of its full therapeutic effect, a certain specific quantity was necessary to be introduced into the system, which quantity was only to be determined by experience.

Dr. Wickersham considered the remedy decidedly productive of injury to the brain when incautiously administered.

Dr. Rauch favored the administration of quinine in small doses, continued for a longer period of time, and thought that the changes in the mode of practice in different localities might be accounted for by changes in the types of diseases. He believed that the remedy was useful in typhoid fever, as a tonic during convalescence, but at no other time during the continuance of that disease.

Dr. Chas. G. Smith considered quinine to be an anti-periodic simply, acting through the medium of the nervous system, and was as appropriate a remedy in one form of periodical disease as in another, hence its efficacy in neuralgia. He did not agree with Headland in his theory that it supplied a deficient element to the blood.

Dr. Bloodgood had commenced in the early portion of his career with the use of small doses of quinine, which he had found entirely successful. Some years afterwards, he, in common with other practitioners in the same locality, had found it necessary to increase the doses in order to produce the desired effect; did not believe the remedy a local irritant; did not approve of small doses, and always waited for a complete intermission before using it at all.

Dr. Graham strongly advocated the use of large doses of quinine; had seen many lives sacrificed by the too sparing use of that remedy. Considered it necessary to place the system thoroughly under its effects, and speedily, in the severer forms of intermittent, in order to insure the life of the patient.

Dr. Hamill had used Carbonate of Ammonia, and also Prussiate of Iron, in conjunction with a diminished portion of quinine, and found them valuable and efficacious substitutes for a portion of the usual quantity of the alkaloid used.

Dr. Ingalls inquired at what periods typhoid fever had been first observed in the West, and in what localities these observations had been made.

Dr. Byford had seen it first in Southern Indiana in 1843.

Dr. Ingalls in Central Illinois in 1847.

Dr. Rauch in Iowa in 1854.

Dr. Bevan had seen cases of typhoid fever fabricated by the abuse of quinine; did not believe that this agent ever originated or kept up intermittent fever, which idea he thought to be an error originating with Hahnneman and his followers.

The debate was continued by Drs. Bloodgood, Graham, Rauch, Orrin Smith, and Ingalls.

Dr. Hay's amendments to the By-Laws proposed at the last meeting were adopted *leviatim*, whereupon the Academy adjourned.

